# Unusually high ratio of shear modulus to Young’s modulus in a nano-structured gyroid metamaterial

**DOI:** 10.1038/s41598-017-10978-8

**Published:** 2017-09-05

**Authors:** Jun-Hyoung Park, Jae-Chul Lee

**Affiliations:** Department of Materials Science and Engineering, Korea University, Seoul, 136-713 South Korea

## Abstract

Unlike the constant nature of elastic coefficients of isotropic bulk materials, the Young’s (E) and shear (μ) moduli of nano-structured (NS) gyroid metamaterials change with relative density (ρ), but at different rates depending on the cell size of the structure. These elastic behaviors displayed by E and μ cause crossover/inversion of these two moduli, such that μ of the NS gyroid metamaterials is greater than E for the structures with ρ < 0.23. This peculiar elastic behavior causes NS gyroid metamaterials to display high μ/E values (~1.0), which are more than 250% larger than the typical values of the bulk material (~0.38), indicating that the NS gyroid metamaterial, even if it is light, is resistant to shear deformation. Here, we report the results of molecular dynamics simulations performed to elucidate the reason for unusually high μ/E values in NS gyroid metamaterials.

## Introduction

In 1967, Luzzati and Spegt^1^ first reported the presence of the single gyriod symmetry in Sr soap surfactant. In 1970, Schoen^2^ introduced an artificial surface, a so-called G-surface (or simply gyroid), in an attempt to design a structure with an infinite periodic minimal surface. This structure was later modeled mathematically by Karcher^3^. Nevertheless, it was not until much later that researchers recognized the existence of the single gyroid structure in nature. The single gyroid structure was discovered in lipid/water systems^1, 4^ and subsequently identified in living organisms such as butterfly wing scales^5–7^ and mitochondria membranes^8, 9^. The structure was also reproduced by the self-assembling characteristics of block copolymers^10, 11^. Extensive studies to unlock the physics underlying the unique properties of this structure/geometry such as its optical^12–14^, electromagnetic^15, 16^, and hydrodynamic properties^17, 18^ are underway through relating the fundamental motif of the gyroid structure to its macroscopic properties. However, the mechanical properties of the gyroid structure have received less attention.

The gyroid structure is lighter and stronger than other structures because it is a triply bi-continuous isotropic structure that fills space with minimal surface. Despite the potential applicability of the gyroid structure in engineering mechanical metamaterials, only a few studies on the mechanical properties of the gyroid structure have been reported. Yan *et al*.^19^ and Bobbert *et al*.^20^ fabricated macroscopic gyroid structures using the selective laser melting technique and measured their stress-strain responses. Khaderi *et al*.^21^ computationally produced a gyroid structure using the finite element method (FEM) to explore its elasto-plastic response. Recently, Lee *et al*.^22^ studied the modulus values of various metamaterials and reported that the Young’s modulus (E) of the modified gyroid metamaterial is as high as 4 times the shear modulus (μ). However, the gyroid structures used in previous studies were characterized by either a large cell size^19, 20, 22^ or a near-zero volume fraction^21^, which differ from the actual nano-structured gyroid materials of living organisms. On this issue, much remains unsettled, thus requiring further analyses.

When a structure/material is reduced to the nano-scale, the fraction of atoms located at the surface (hereinafter, referred to as “surface atoms”) increases. Surface atoms are less effective in carrying a tensile load due to their comparatively weak atomic bonding (equivalently, high energy state) and are thus expected to contribute less to E. FE analysis (employed in previous studies) is a continuum-based large-scale simulation technique for analyzing the mechanical properties of materials and typically does not take into account the surface effect in nano-scale structures. Hence, FE analysis overestimates the E value and thus, in many cases, is inappropriate for studying the structure-property relationships of nano-structured gyroid metamaterials. Of various simulation techniques, molecular dynamics (MD) simulation, owing to its ability to depict atomic-scale structures and to evaluate the corresponding mechanical properties, can explain the structure-property relationships of nano-structured materials that cannot be obtained via FE simulations and experimental approaches.

The present study aims to evaluate the elastic properties of nano-structured Al with a single gyroid structure (hereinafter referred to as NS gyroid Al) and to analyze their anomalous behaviors by incorporating the surface energy of the structure using classical molecular dynamics (MD) simulations. Unlike the constant nature of the elastic coefficients of the bulk isotropic material, the values of E and μ of the NS gyroid metamaterial change with relative density, but at different rates depending on the cell size of the structure. The different behaviors displayed by E and μ cause the NS gyroid metamaterial to display unusually high μ/E values (~1.0), which is greater by more than 250%, compared to the typical values (~0.38) of the bulk material. This unique elastic behavior was explained based on MD simulations by quantifying the different effects that the surface atoms have on E and μ.

## Results and Discussion

### Preparation of NS gyroid Al

**Figure 1:**
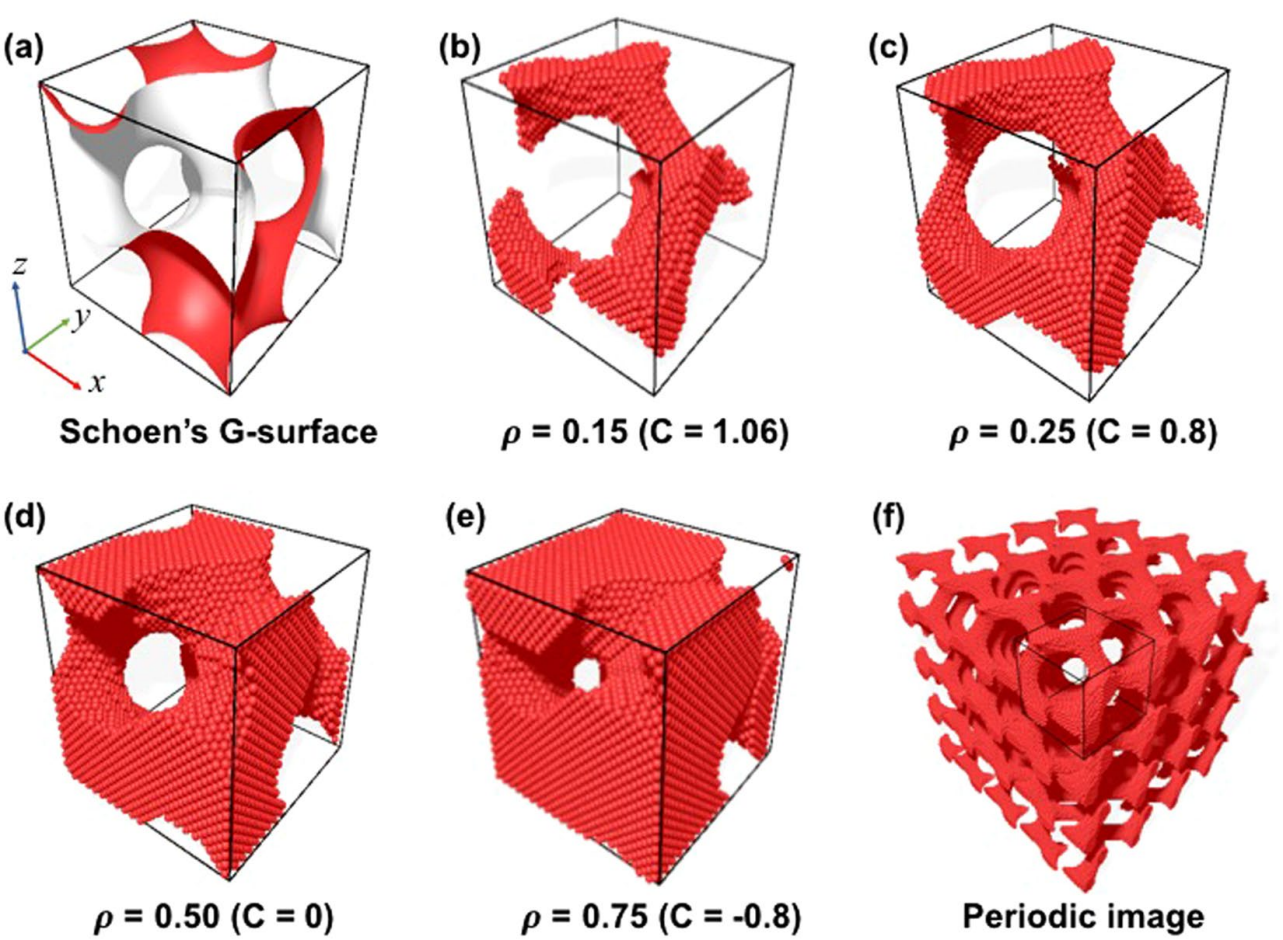
(**a**) Unit cell of the Schoen’s G-surface with C = 0 and L = 8.1 nm. Images of unit cells of gyroid structures with differing relative densities (${\rm{\rho }}$) of **(b)** 0.15, **(c)** 0.25, **(d)** 0.50, and **(e)** 0.75. **(f)** Bicontinuous gyroid metamaterial constructed for mechanical tests by applying the periodic boundary condition.

To construct the NS gyroid Al, a single gyroid level surface was prepared using the unit cell of the Schoen’s G-surface (Fig. 1a) given by

1 $$\sin (2{\rm{\pi }}\frac{{\rm{x}}}{{\rm{L}}})\cdot \,\cos (2{\rm{\pi }}\frac{{\rm{y}}}{{\rm{L}}})+\,\sin (2{\rm{\pi }}\frac{{\rm{y}}}{{\rm{L}}})\cdot \,\cos (2{\rm{\pi }}\frac{{\rm{z}}}{{\rm{L}}})+\,\sin (2{\rm{\pi }}\frac{{\rm{z}}}{{\rm{L}}})\cdot \,\cos (2{\rm{\pi }}\frac{{\rm{x}}}{{\rm{L}}})={\rm{C}},\,$$

where L is the unit-cell length of the single gyroid structure and C is the threshold of the level surface that can convert the gyroid minimal surface to a three-dimensional solid network with a finite volume. This determines the relative density (ρ, also called the volume fraction), defined by the ratio of the volume enclosed by the gyroid level surface to that of the gyroid unit cell. Unit cells of various NS gyroid structures (with different relative densities) were prepared by changing the values of L and C in Eq. (1). For given values of L and C, the atoms that did not satisfy Eq. (1) were selectively eliminated from the perfect Al crystal cube (with the <100> direction parallel to the x-axis) to produce NS gyroid metamaterials with different relative densities (Fig. 1b–e). The minimum ${\rm{\rho }}$ value of the gyroid structure was taken to be 0.15 to avoid collapse of the gyroid structures due to the disconnection of struts, whereas the maximum ${\rm{\rho }}$ value was selected as 0.75, which is slightly smaller than the maximum possible value of 0.80 in triply periodic, bicontinuous structures^23^. Periodic boundary conditions were imposed along the x-, y- and z-axes to prepare the supercell structures of the gyroid metamaterials needed to evaluate the elastic properties (Fig. 1f).

### Geometric characteristics of the gyroid structure

The gyroid metamaterial is a bicontinuous structure characterized by a triply periodic network of identically shaped solid phase and pores. If the properties (E and μ) measured from this structure represent the entire structure, the external load imposed on the structure should create a homogeneous deformation over the entire volume of the structure. To attain such deformation conditions, the area fractions occupied by the material should be the same over all the cross-sections parallel to the loading direction. Therefore, before calculating the E and μ values of the gyroid metamaterial, the geometrical isotropy of the gyroid structure was tested in the following by calculating the area fractions of arbitrarily chosen cross-sections of the gyroid cell.

**Figure 2:**
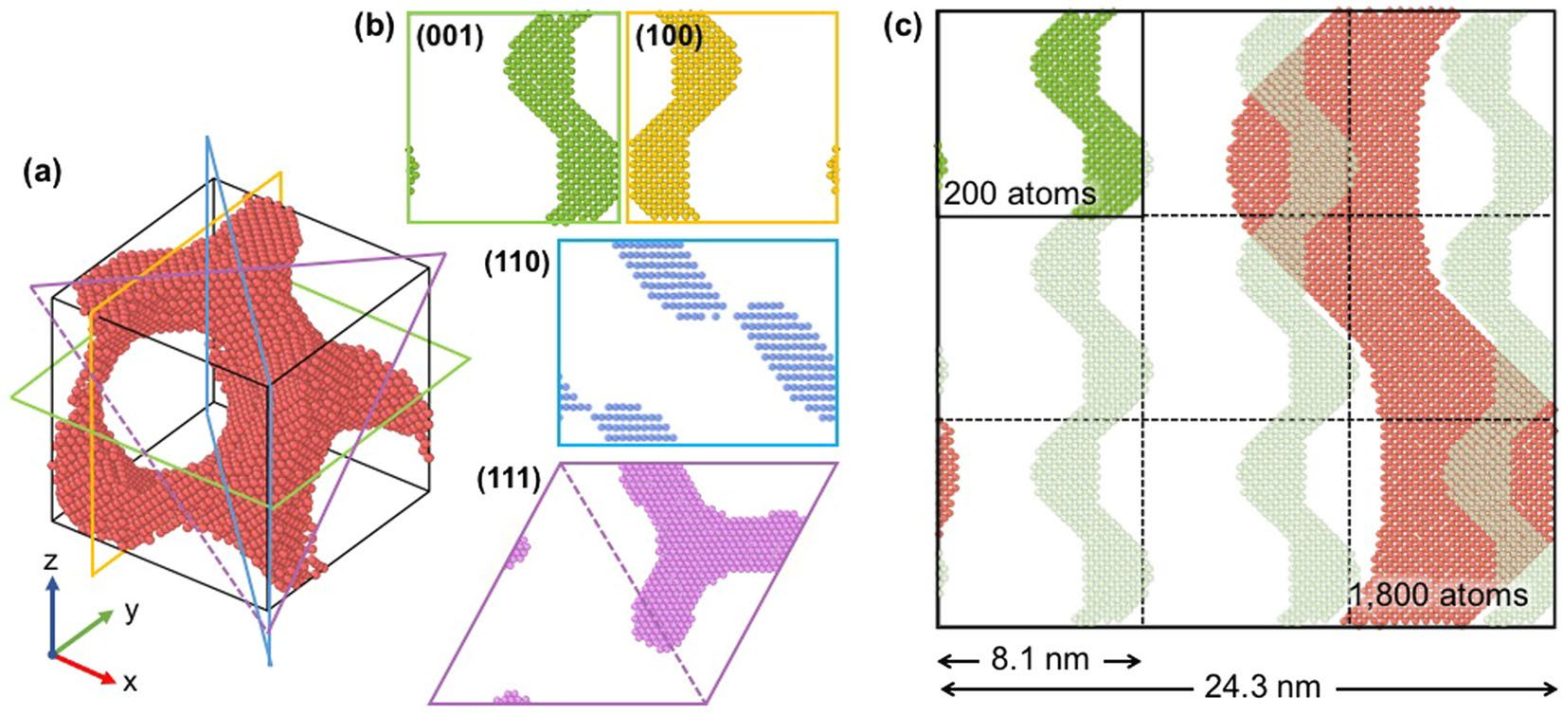
(**a**) A gyroid unit cell (L = 8.1 nm) and **(b)** various cross-sections viewed from the planes parallel to (001), (100), (110), and (111) of the cell, showing that the area fractions occupied by constituent atoms are the same regardless of the plane. In the present case, all shaded areas are composed of 200 atoms that occupy 25% of the section. **(c)** Comparison of the cross-sections of the 8.1 and 24.3 nm gyroid unit cells, showing that the area fraction obtained from the 24.3 nm gyroid cell is the same as that occupied by the cross-sections of 9 individual gyroid cells with L = 8.1 nm.

Figure 2a shows the unit cell of the gyroid structure with L = 8.1 nm and ${\rm{\rho }}$ = 0.25, whereas Fig. 2b displays various cross-sectional shapes viewed from planes parallel to (001), (100), (110), and (111) of the gyroid unit cell. The area fractions occupied by atoms are approximately the same (~25%) for all cross-sections. The same feature holds for any cross-section regardless of the cell size. Figure 2c is an example, showing that the area fractions occupied by the atoms in a plane parallel to (100) are the same (25%) even if the cell sizes of the single gyroid structures are different (L = 8.1 and 24.3 nm). The present analyses indicate that the numbers of Al atoms (i.e., the area fraction) that participate in the deformation are the same for all planes normal/parallel to the loading direction. Therefore, the E and μ values evaluated from the gyroid metamaterial represent the entire volume of the structure, suggesting that the gyroid structure is geometrically isotropic. However, when viewed from a crystallography perspective, the gyroid model starts from a single crystalline Al with its <100> direction parallel to the x-axis and thus is anisotropic. However, the anisotropic ratio ( = 2C_44_/(C_11_–C_12_) in Voigt notation) is very small ( = 1.22) for Al. Therefore, the model is much approximated to be isotropic for the whole cell.

### Crossover behavior of the NS gyroid metamaterial

**Figure 3:**
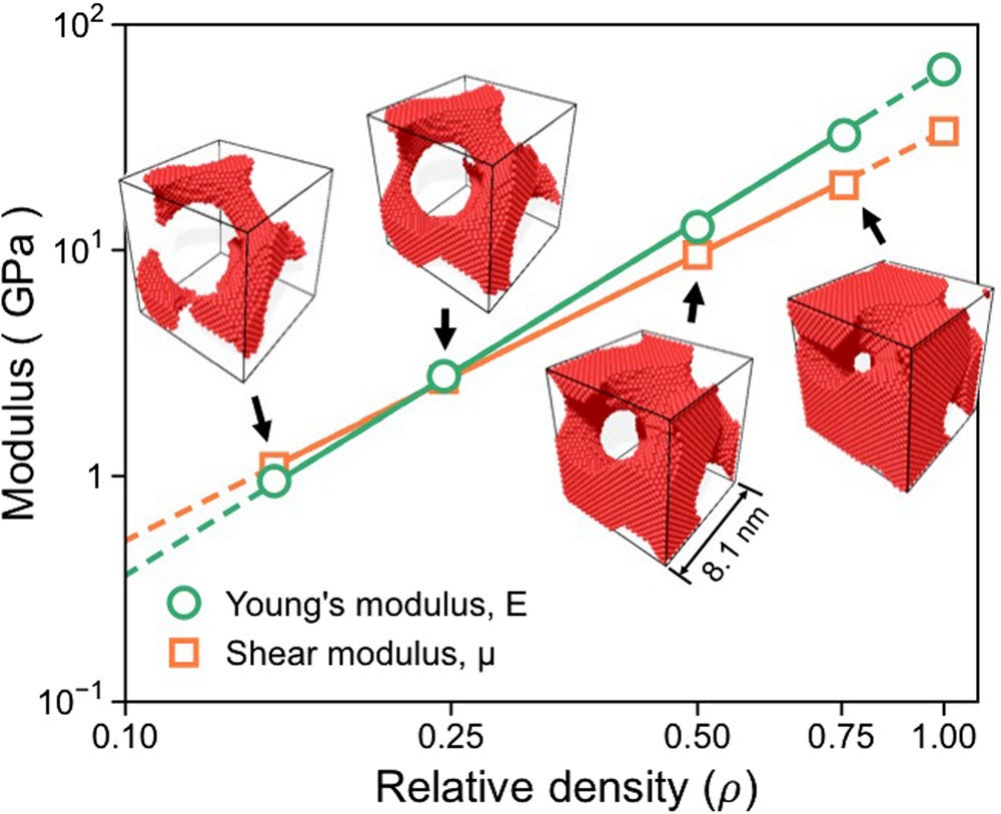
Changes in the values of E and μ evaluated as a function of the relative density of the NS gyroid Al with a cell size of 8.1 nm. Note: in this particular NS gyroid Al with L = 8.1 nm, the μ value is greater than that of E when ${\rm{\rho }}\,$< 0.23.

MD simulations were performed on the NS gyroid Al with a unit-cell length of 8.1 nm to explore the dependence of E and μ on the relative density (${\rm{\rho }}$). As shown in Fig. 3, although both the E and μ values of the NS gyroid Al (L = 8.1 nm) decrease with decreasing ${\rm{\rho }}$, their rates are different; E changes in proportion to ${{\rm{\rho }}}^{2.2}$ whereas μ changes according to ${{\rm{\rho }}}^{1.8}$. The results indicate that as ${\rm{\rho }}$ decreases, E decreases faster than μ. The different sensitivities of E and μ to ${\rm{\rho }}$ of the NS gyroid Al cause inversion/crossover of these two moduli such that μ is greater than E in structures with ${\rm{\rho }}\,$< 0.23. Considering that E of the isotropic bulk material is nearly three times the μ value (as per the relationship $={\rm{E}}/2(1+{\rm{\nu }})$), the crossover behavior displayed by the NS gyroid metamaterial is unusual and unexpected.

### Effects of ρ and L of the gyroid cell on the μ/E ratio

**Figure 4:**
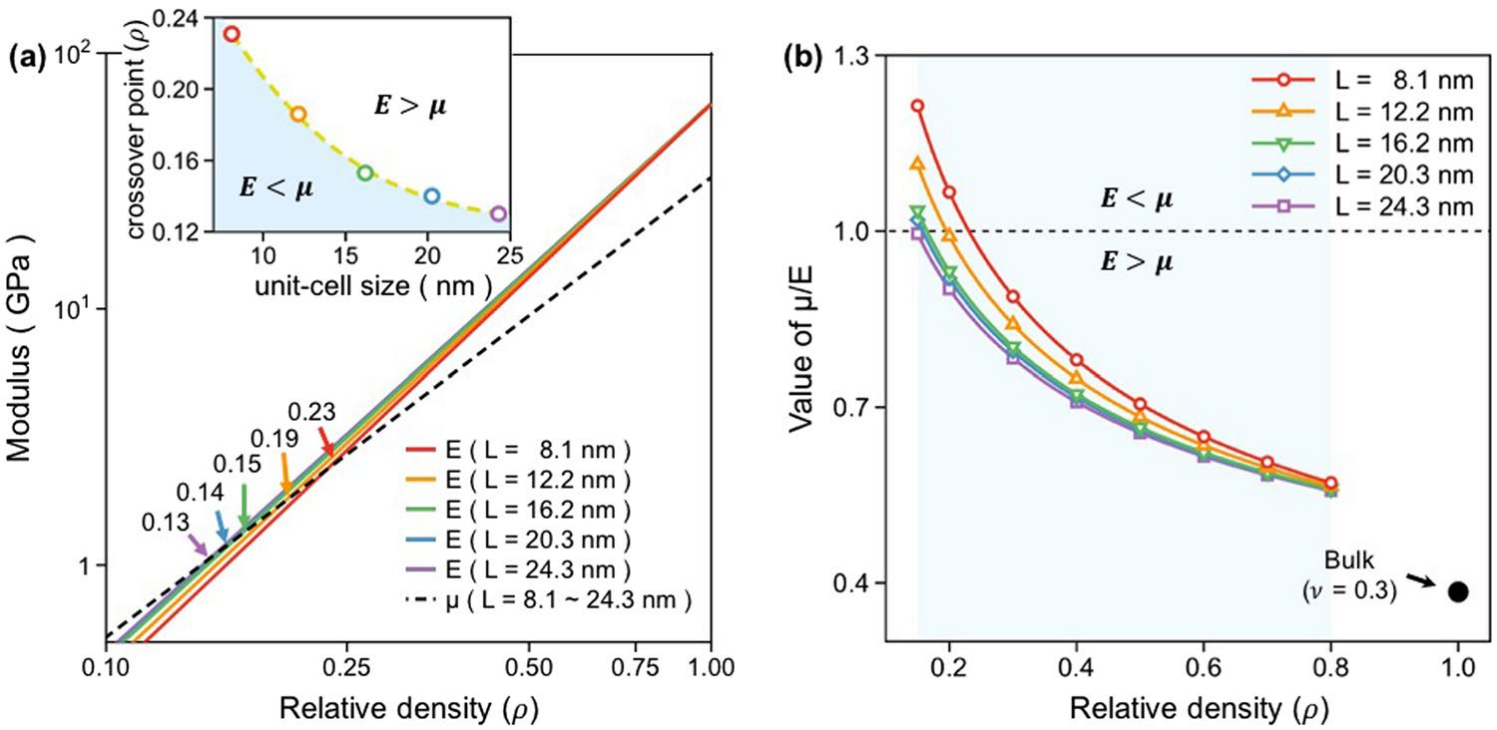
(**a**) Variations in the values of E and μ evaluated as a function of the relative density of the NS gyroid Al with various cell sizes. The inset of (**a**) shows the changes in the crossover point determined as a function of the unit-cell size of the NS gyroid Al. **(b)** Variations in the value of μ/E evaluated as a function of the relative density of the NS gyroid Al with various cell sizes.

In addition to the ${\rm{\rho }}$ value of the NS gyroid Al, the cell size (L) of the gyroid structure is another geometric parameter that influences the modulus. This is because the fraction of surface atoms comprising the NS gyroid Al increases as the cell size decreases. Figure 4a shows changes in E and μ of the NS gyroid Al calculated as a function of ${\rm{\rho }}$ and L. Two distinct features are noted; although values of both E and μ decrease with decreasing ${\rm{\rho }}$, the rates of decrease of E and μ differ depending on the cell size. The decrease rate of E is sensitive to the cell size; it is faster in structures with smaller cell size. The decrease rate of μ is nearly unaffected by the cell size. The different sensitivities of E and μ to the cell size cause the crossover point to shift toward higher ${\rm{\rho }}$-values as the cell size decreases (see the inset of Fig. 4a). This peculiar elastic behavior displayed by E and μ causes the NS gyroid metamaterial to reveal μ/E values (≥1.0) substantially greater than the typical value (~0.38) displayed by the bulk material (Fig. 4b). This large value of μ/E was not observed from other bicontinuous structures such as the Schwarz D (diamond), P (primitive), and F23 structures because they do not display the crossover/inversion of two moduli (see Supplementary Fig. S1). As such, we consider the large value of μ/E to be the characteristics property specific to the single gyroid metamaterial.

The present finding on the elastic behavior of the NS gyroid Al characterized by high μ/Ε values implies that the NS gyroid metamaterial, even if it is light, can better resist specific motions that create shear stress. Considering that the NS gyroid metamaterial is characterized by large surface area (on which atoms are loosely bonded), this anomalous elastic behavior of the NS gyroid metamaterial must be related to the surface effect associated with weak atomic bonding. The physics of the current observation is explained by clarifying the different effects that the surface atoms have on E and μ of the NS gyroid Al.

### Surface atoms and their effect on E of the gyroid metamaterial

Having identified the different dependencies of E and μ on the cell size of the NS gyroid metamaterial (see Fig. 4a), we next elucidate why these two properties behave differently with changing cell size. The peculiar behaviors of E and μ discussed in Fig. 4a were absent in previous studies based on FE analyses^21^ because the model employed for FE calculations overlooked surface effects. The puzzle observed in the current study is explained by quantifying the surface effect that influences E and μ of the NS gyroid structures with various cell sizes. To quantitatively evaluate the surface effect of the NS gyroid metamaterial on E, we first defined the fraction of surface atoms (${\rho }_{{\rm{s}}}$) that do not contribute to E of the NS gyroid metamaterial using

2 $${{\rm{\rho }}}_{{\rm{s}}}={\rm{\rho }}-{{\rm{\rho }}}_{{\rm{e}}}.$$

where ρ is the “apparent” relative density of the gyroid structure and ${\rho }_{{\rm{e}}}$ is the “effective” relative density that actually contributes to the modulus. Since the value of ${\rho }_{{\rm{s}}}$ quantifies the surface effect, it varies with values of L and ρ of the gyroid structure and thus can be determined by simultaneously considering these geometric parameters.

**Figure 5:**
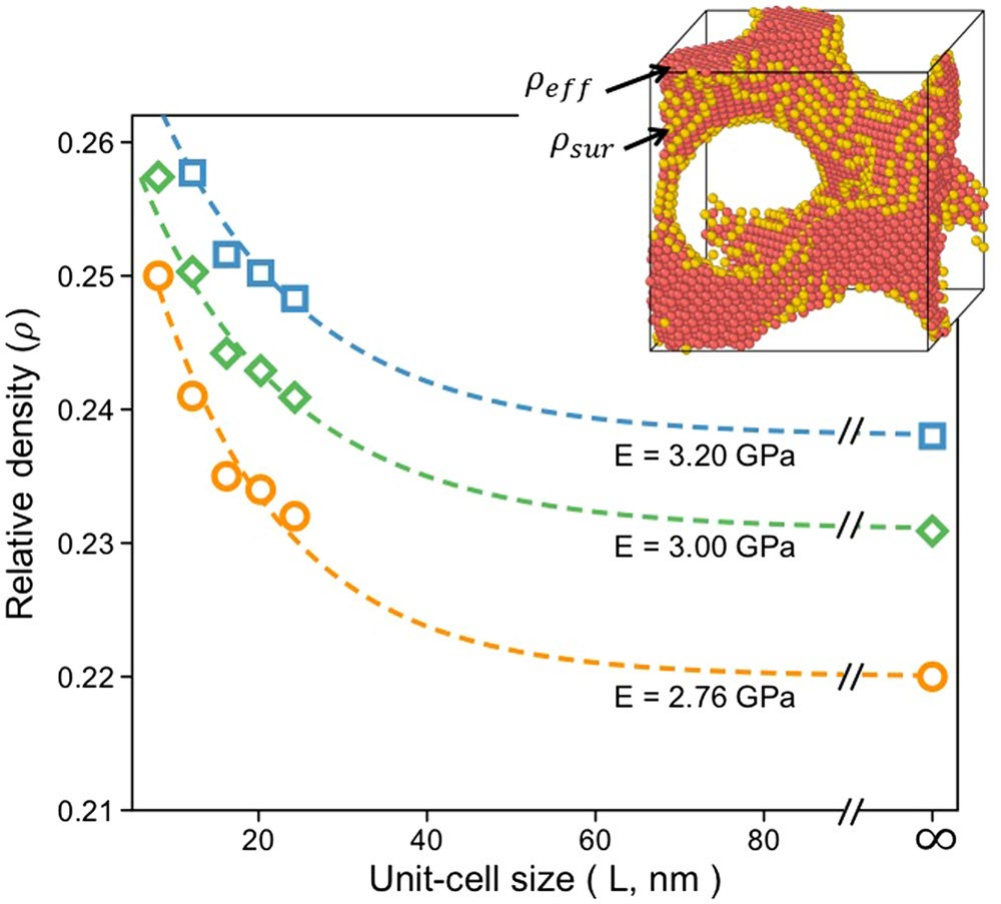
Variations in E evaluated as a function of the cell size of NS gyroid Al with different relative densities.

Figure 5 shows the traces of the sets of L and ρ required for the NS gyroid Al to display certain predetermined E values. For each curve in Fig. 5, the ρ value of the gyroid Al with a large cell size (L = ∞) can be regarded as ${\rho }_{{\rm{e}}}$ because the surface effect can be ignored for the structure with a large cell size. In the case of the gyroid Al with E = 2.76 GPa, the value of ${\rho }_{{\rm{e}}}$ is $0.22$. However, as the cell size is reduced, the value required to obtain the same E value increases to compensate for the increased amount of surface atoms that do not contribute to E (owing to their loose atomic bonding). The quantification of surface atoms in the NS gyroid Al is therefore possible by determining the increment in relative density (Δρ). For example, to prepare the NS gyroid Al with L = 8.1 nm and E = 2.76 GPa, the relative density of the structure should be 0.25 (see the curve corresponding to E = 2.76 GPa in Fig. 5). This value is greater than that of the large-scale gyroid structure (${\rho }_{{\rm{e}}}$ 0.22) by 0.03, which is equivalent to the fraction of atoms that do not contribute to E and thus, is equal to ${\rho }_{{\rm{s}}}$. The present result suggests that E, which to date is known to be dependent primarily on the orientation of a given bulk crystal, can vary with cell size when the structure is reduced to the nano-scale.

### Resistance of surface atoms to shear deformation and its effect on μ

As opposed to the cell-size-dependent characteristics of E, the μ value of the NS gyroid metamaterial is nearly unaffected by cell size (Fig. 4a), indicating that μ is less sensitive to the surface effect. This difference in the characteristics of E and μ arises from differences in the modes of atomic motion under the action of uniaxial tension and simple shear. In the case of uniaxial tension, the weak atomic bonding of the surface atoms of the NS gyroid Al causes a decrease in the resistance to tensile deformation, which in turn reduces E. In the meantime, shear deformation is characterized by the sliding action of one atomic layer over the next. When simple shear deformation is viewed from an atomic-scale perspective, atoms that move over the reference/neighboring plane would slide around the reference atoms maintaining a constant interatomic spacing. Therefore, unlike the case of uniaxial tension, the increased interatomic spacing associated with the weak atomic bonding of surface atoms would offer a resistance to the sliding of atoms, which is revealed by the increase in potential energy. This scenario may serve as the basis for the insensitivity of μ to the cell size of the gyroid structure, which is tested in detail by calculating changes in the potential energy of the surface plane/atom during sliding over the reference plane.

**Figure 6:**
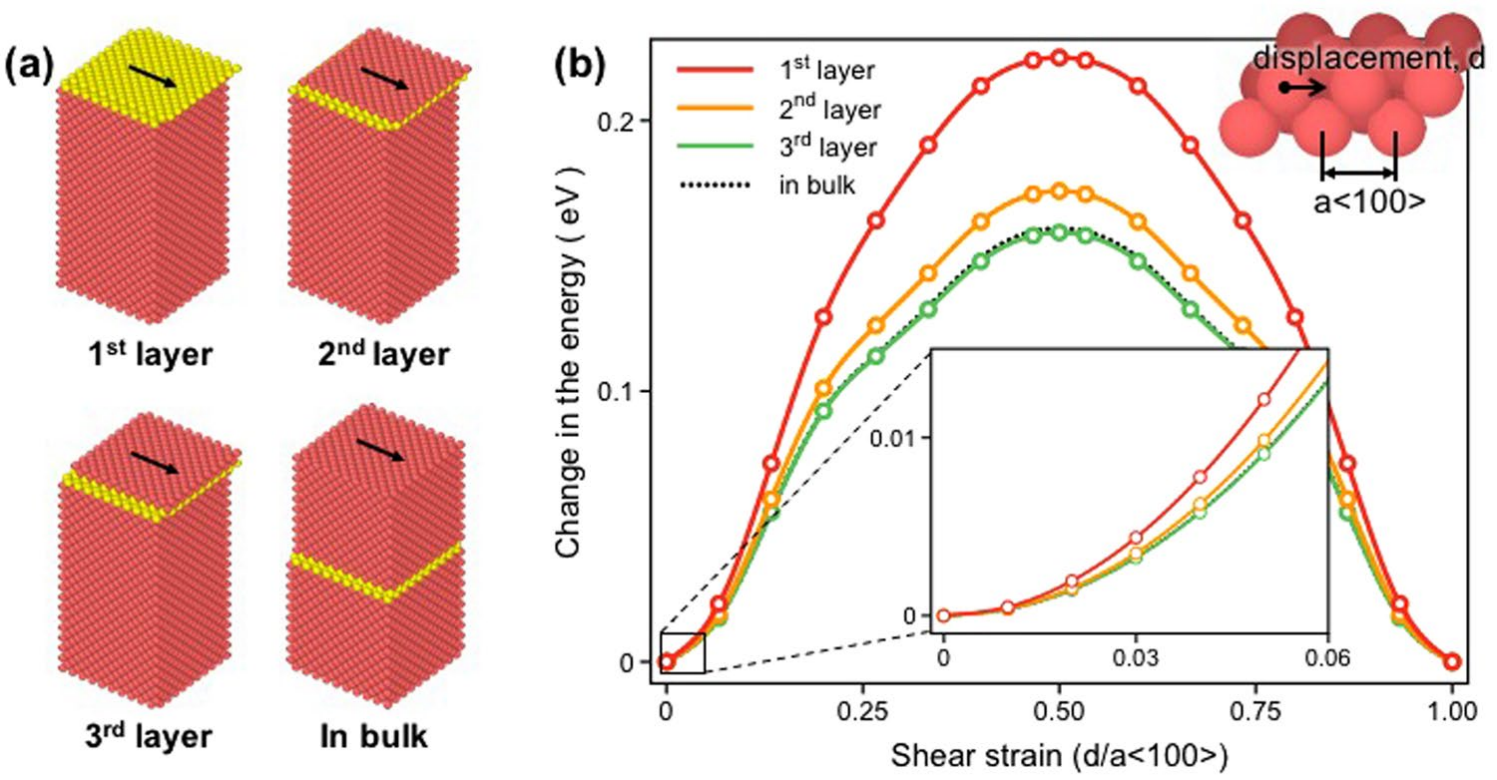
(**a**) MD models of the Al single crystal used to calculate changes in the potential energy of the Al atom by moving it along <100 > in (100). Two atomic layers colored in yellow denoted the shearing and the reference plane that are located at different depths from the free surface. **(b)** Changes in the potential energy of Al atoms located in different (100) layers relative to the free surface. The energy was calculated by moving the Al atom by one lattice parameter (*a* = 4.05 Å) along <100 > of the reference plane.

Figure 6 shows changes in the potential energy of the Al atom calculated by moving the (100) layers of the surface region over the neighboring/reference (100) layer by one lattice parameter (*a* = 4.05 Å) along <100> . Despite the weak bonding of the surface atoms, higher energy is required for shear deformation as the atom is located close to the free surface. However, no measurable change is observed in the energy of deformation if the atomic layers are located more than three layers (a few Å) away from the free surface (Fig. 6b). When the NS gyroid Al is subjected to shear deformation within the elastic range (γ = 0.03 in the present study), the energy required for shear deformation is nearly constant regardless of the location of atoms (see the inset of Fig. 6b). This analysis explains why the μ value of the gyroid metamaterial is virtually unaffected, though the fraction of surface atoms increases with decreasing cell size. Rather, as was previously discussed in Fig. 4a, the μ value is determined only by the number of atoms in the shearing plane, which in the case of a gyroid metamaterial is the same as the relative density of the gyroid unit cell.

In summary, the NS gyroid Al displays unusually high μ/E values (≥1.0) that are significantly greater than the typical value (~0.38) of the bulk material. This characteristic of the NS gyroid Al, even if it is light, makes the material more resistant to shear deformation. NS gyroid metamaterials are considered suitable for building micromechanical systems where shear stress prevails under loading conditions. Systematic future work is necessary on this issue.

## Conclusions

MD simulations performed on the NS gyroid metamaterial showed that values of both E and μ decrease with decreasing relative density of the gyroid structure. However, the rates of decrease of E and μ differ depending on the cell size of the structure. E is sensitive to decreasing cell size, whereas μ is observed to be nearly unaffected by cell size. The different sensitivities of E and μ to the relative density and cell size of the NS gyroid Al lead to an inversion of these two moduli; the NS gyroid Al displays unusually high μ/E values (≥1.0) that is greater by more than 250% compared to the typical value (~0.38) of the bulk material. This characteristic of the NS gyroid Al makes the material more resistant to shear deformation.

High μ/E values of the NS gyroid metamaterial arise mainly from the different effects of the surface atoms on the E and μ values. The surface atoms, owing to their weak atomic bonding, reduce the resistance to tensile deformation with decreasing relative density and cell size, thereby lowering the E value of the structure. On the other hand, the weak atomic bonding of surface atoms causes their interatomic spacing to increase and acts as a resistance to shear deformation. However, the μ value is nearly unaffected by the cell size of the gyroid structure because the fraction of surface atoms is usually small ( <0.03) and the energy required for shear deformation is nearly unchanged regardless of the location of atoms in terms of distance from the free surface.

## Methods

### Evaluation of elastic properties

The embedded atom method (EAM) potential developed by Mishin *et al*.^24^ was employed to prepare the Al crystal (for the choice of potential used in this study, see Supplementary Figs. S2 and S3). Although the EAM potential used for MD simulations has already been extensively validated against a large set of experimental properties and *ab initio* data, the reliability of the potential and the suitability of the calculation methods in this study were tested again by evaluating the stacking fault energy (SFE) of pure Al and the Young’s modulus of mono-crystalline Al oriented along <100> ; the SFE was evaluated by calculating the general stacking fault energy (GSFE) curve. Of all potentials tested^24–27^, the EAM potential developed by Mishin *et al*.^24^ produces the SFE similar to that measured from experiments (~150 mJ/m^2^). In addition, the Young’s modulus calculated from the computationally generated <100> Al is 63.3 GPa, which is similar to the experimental value (63.2 GPa) measured along <100> ^28^. This ensures the validity of the potential and the calculation procedures employed to evaluate the elastic properties of the NS gyroid metamaterial used in this study.

Prior to mechanical tests of the NS gyroid Al, each structure was relaxed using conjugate gradient energy minimization and subsequently equilibrated using a Nosé-Hoover thermostat at 300 K for 5 ns in a large-scale atomic/molecular massively parallel simulator (LAMMPS)^29^. To study the effect of the geometric variables of the NS gyroid Al on the E and μ values, we calculated the stress-strain curves of the NS gyroid Al with various values of ${\rm{\rho }}$ and L by applying uniaxial tension and simple shear along the z- and xy-directions, respectively, (see Fig. 1a) at the strain rate of $1\times {10}^{7}{s}^{-1}$. Both tensile and shear tests of the computationally generated NS gyroid Al were performed by maintaining a uniform strain loading condition. This was achieved by linearly varying the velocity (applied to the individual atoms along the loading direction) from zero at the fixed end to a maximum value (corresponding to ε and γ = 0.03) at the loading end. The values of E and μ of the NS gyroid Al were obtained from the slope in the stress-strain curve measured during testing of each NS gyroid Al (for example plots used to determine E and μ, see Supplementary Fig. S4).

## Electronic supplementary material

Supplementary Information
