# Supplementary material for: Unusually high ratio of shear modulus to Young’s modulus in a nano-structured gyroid metamaterial
Source: Sci Rep. 2017 Sep 5;7:10533. doi: 10.1038/s41598-017-10978-8 (PMC5585398; doi:10.1038/s41598-017-10978-8)
Supplement: Supplementary file 1 — Supplementary Information [file 41598_2017_10978_MOESM1_ESM.pdf]

Supplementary Information

Unusually high ratio of shear modulus to Young's modulus in a nano-structured gyroid metamaterial

Jun-Hyoung Park and Jae-Chul Lee*

Department of Materials Science and Engineering, Korea University, Seoul 136-713, South Korea

*To whom correspond should be addressed, E-mail: jclee001@korea.ac.kr

1. Calculations of E and μ values for various bicontinuous structures with a triply periodic minimal surface

The E and μ values were calculated for various bicontinuous structures, such as the diamond (D), primitive (P), and F23 structures, with a triply periodic minimal surface. First, the unit cells of the bicontinuous structures were prepared using Eqs. (S1-S3) and are shown in Fig. S1a. The periodic boundary condition was applied to each cell to prepare computational bicontinuous metamaterials for mechanical tests using MD. The structures were then deformed to evaluate the E and μ values (for details, see Methods). It was found from Figs. S1b-d that all bicontinuous structures other than the single gyroid structure does not show the crossover/inversion of two moduli, causing them to display comparatively low μ/E values.

Diamond (D) structure: $S_x^1 S_y^1 S_z^1 + S_x^1 C_y^1 C_z^1 + C_x^1 S_y^1 C_z^1 + C_x^1 C_y^1 S_z^1 = C$ (S1)

Primitive (P) structure: $-(C_x^1 + C_y^1 + C_z^1) = C$ (S2)

F23 structure: $8C_x^1 C_y^1 C_z^1 - 8S_x^1 C_y^1 C_z^1 - 2(C_x^2 + C_y^2 + C_z^2) = C$ (S3)

Here, we simplify trigonometric functions as $S_\alpha^n = \sin\left(2n\pi\frac{\alpha}{L}\right)$ and $C_\alpha^n = \cos\left(2n\pi\frac{\alpha}{L}\right)$, where L is the cubic unit cell length and C is the threshold of level surface.

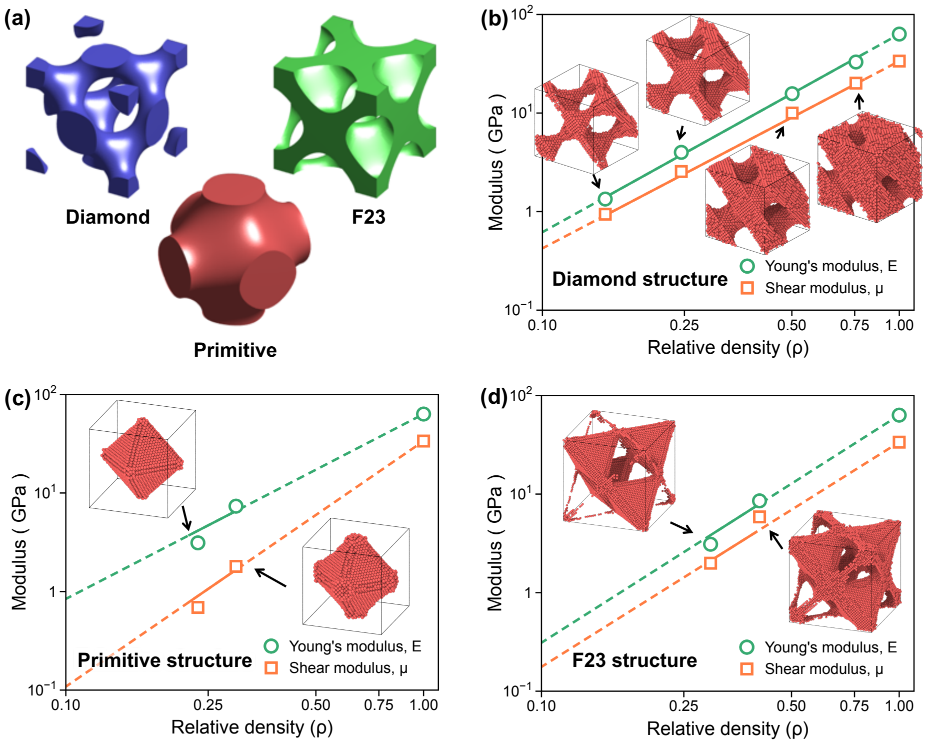

Figure S1. (a) Unit cells of the bicontinuous cubic network of diamond (D), primitive (P), and F23 structures. **(b-d)** Changes in the values of E and μ evaluated as a function of the relative density of **(b)** diamond, **(c)** primitive, and **(d)** F23 structures. Note that the unit cell length is 8.1 nm for all bicontinuous structures. The E values of these structures are greater by more than 150%, compared to the μ values.

2. Evaluation of the potentials employed for MD simulations

The validity of the potentials used for MD simulations was tested by calculating the stacking fault energy (SFE, ~150 mJ/m^2) of an Al crystal. The values of the SFE were obtained from the generalized stacking fault energy curves calculated using potentials based on EAM[1,2], RFMEAM[3], and ReaxFF[4] (Fig. S2). Of the tested potentials, the potential developed by Mishin et al.[1] best describes the SFE of the Al crystal. The SFEs predicted by other potentials[2-4] are either too large or too small, occasionally leading to the prediction of erroneous deformation processes. Therefore, they were unable to produce reliable mechanical responses.

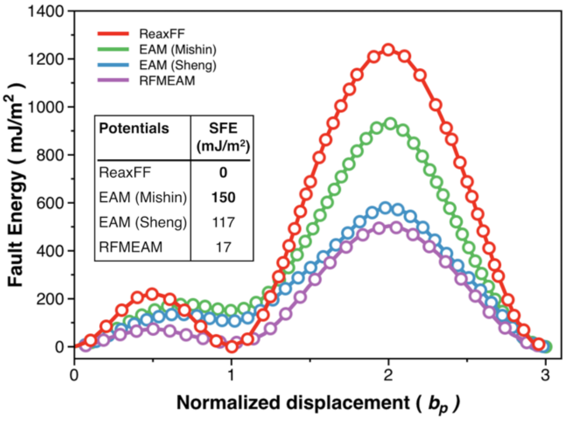

Figure S2. Fault energy curves calculated for a perfect Al crystal.

The suitability of the EAM potential (developed by Mishin et al.) for predicting the mechanical responses of an Al single crystal was tested by computing the stress-strain curves. Figure S3 shows the stress-strain curve predicted by MD simulations, superimposed with that obtained from *in situ* TEM tensile tests previously performed by the Kim et al.[5]. It is clear that the values of the yield strength (3.2-3.5 GPa), elastic limit (4.6-5.0%), and Young's modulus (~70 GPa) of the defect-free <110> Al crystal evaluated by both methods agree with each other. We consider the potential developed by Mishin et al. more reliable than the other potentials tested in the study for predicting the mechanical response.

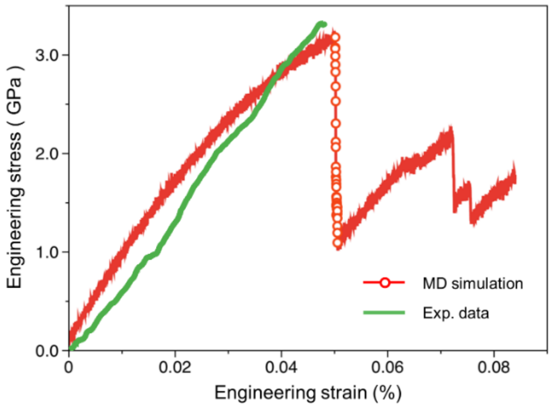

Figure S3. Stress-strain curve of the <110> Al crystal obtained from MD simulations based on the EAM potential developed by Mishin et al.. Superimposed is the stress-strain curve of a defect-free <110> Al nanowire measured using micro-mechanical testing based on *in situ* transmission electron microscopy.

3. Evaluation of E and μ of a NS gyroid Al using MD simulations

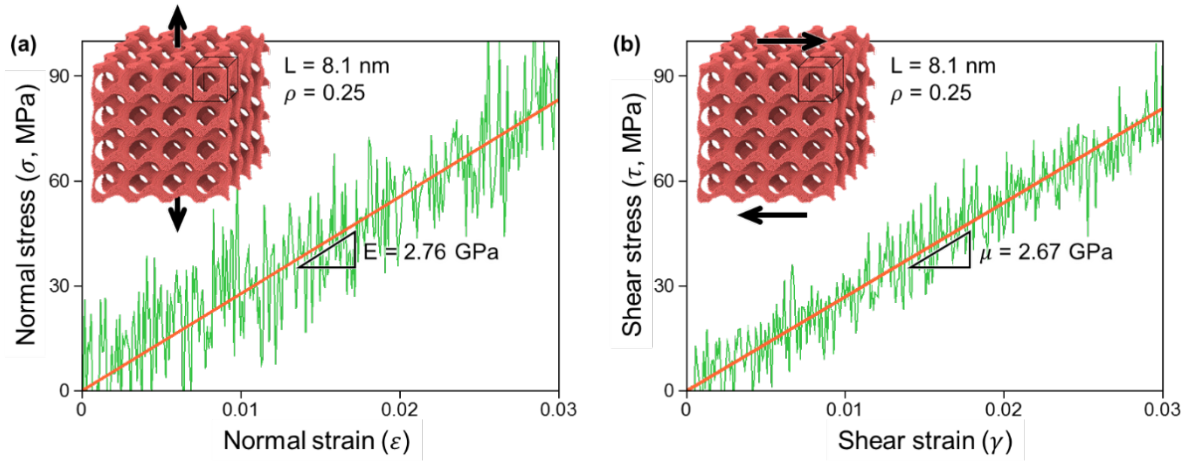

Figure S4. Stress-strain curves of the NS gyroid Al (L = 8.1 nm and ρ = 0.25) obtained from MD simulations (denoted in green) under **(a)** uniaxial tension and **(b)** simple shear. The lines denoted in orange are the best-fit lines used to measure the Young's modulus and the shear modulus.

References

1 Mishin, Y., Farkas, D., Mehl, M. & Papaconstantopoulos, D. Interatomic potentials for monoatomic metals from experimental data and ab initio calculations. *Physical Review B* **59**, 3393 (1999).

2 Sheng, H., Kramer, M., Cadien, A., Fujita, T. & Chen, M. Highly optimized embedded-atom-method potentials for fourteen fcc metals. *Physical Review B* **83**, 134118 (2011).

3 Lazić, I. & Thijsse, B. J. An improved molecular dynamics potential for the Al–O system. *Computational Materials Science* **53**, 483-492 (2012).

4 Hong, S. & van Duin, A. C. Molecular dynamics simulations of the oxidation of aluminum nanoparticles using the ReaxFF reactive force field. *The Journal of Physical Chemistry C* **119**, 17876-17886 (2015).

5 Kim, S. H. *et al.* Tensile test of an Al nanowire using in-situ transmission electron microscopy and its dynamic deformation behavior. *Journal of Korean Institute of Metals and Materials* **54**, 386-389 (2016).
